# Identification of transforming growth factor beta induced (TGFBI) as an immune-related prognostic factor in clear cell renal cell carcinoma (ccRCC)

**DOI:** 10.18632/aging.103153

**Published:** 2020-05-14

**Authors:** Guo-Wei Du, Xin Yan, Zhao Chen, Ren-Jie Zhang, Kurerban Tuoheti, Xiao-Jie Bai, Hua-Hui Wu, Tong-Zu Liu

**Affiliations:** 1 Department of Urology, Zhongnan Hospital of Wuhan University, Wuhan 430071, China

**Keywords:** clear cell renal cell carcinoma, prognostic biomarkers, tumor immune microenvironment, copy number variations, DNA methylation

## Abstract

Clear cell renal cell carcinoma (ccRCC) is the most common subtype among kidney cancer, which has poor prognosis. The aim of this study was to screen out novel prognostic biomarkers and therapeutic targets for immunotherapy, and some novel molecule drugs for ccRCC treatment. Immune scores ranged from -1109.36 to 2920.81 and stromal scores ranged from -1530.11 to 1955.39 were firstly calculated by applying ESTIMATE algorithm. Then 17 DEGs associated with immune score and stromal score were further identified. 6 candidate hub genes were screened out by performing overall survival (OS) and disease-free survival analyses based on TCGA-KIRC data, one of which including TGFBI was further regarded as hub gene associated with prognosis by calculating the R^2^ (R^2^ = 0.011, *P* = 0.018) and AUC (AUC = 0.874). The prognostic value of TGFBI was validated by performing OS, CSS, and PFS analyses based on GSE29609 and E-MTAB-3267. CMap analysis suggested that 3 molecule drugs might be novel choice for ccRCC treatment. Further analysis demonstrated that CNVs of TGFBI was associated with OS of patients with ccRCC. TGFBI expression was also correlated with histologic grade, pathologic stage, and immune infiltration level, significantly. TGFBI was the most relevant gene with OS among the candidate hub genes, which might be novel DNA methylation biomarkers for ccRCC. In conclusion, our findings indicated that TGFBI was correlated with prognosis of patients with ccRCC, which might be novel prognostic biomarkers, and targets for immunotherapy in ccRCC. Three small molecule drugs were also identified, which showed strong potential for ccRCC treatment.

## INTRODUCTION

Immunotherapy is one of the treatment methods for malignant tumors at present, which mainly uses the immune effects of autoimmune or alloimmune cells in patients to improve the symptoms, prolong the survival and improve the prognosis [1, 2]. In recent years, immunotherapy has become a novel treatment for cancers, whose effectiveness and safety have been gradually conformed [3]. With the development of precision medicine and immunotherapy for cancers, nowadays, more and more researchers focus on finding out more accurate therapeutic targets for immune treatment [4, 5].

Kidney cancer is one of the most common malignances around the world, which is with poor prognosis [6]. According to recent statistics from the International Agency for Research on Cancer (IARC), part of the World Health Organization (WHO), there were 403,262 new cases of kidney cancer and 175,098 associated deaths worldwide in 2018. Renal cell carcinoma (RCC) accounted for approximately 90% of kidney cancers, the commonest histological subtype of which was clear cell renal cell carcinoma (ccRCC) [7, 8]. Although surgical treatment was the most effective therapy for localized ccRCC, there was a lack of drugs for adjuvant treatment [9]. What was worse, there was no effective treatment method for advanced ccRCC [10]. Thus, in this study, we tried to find out some novel prognostic biomarkers, which might be novel targets for immunotherapy.

For the first time, in this study, we firstly calculated immune score and stromal score of each case from TCGA-KIRC data, by applying Estimation of Stromal and Immune cells in Malignant Tumor tissues using Expression data (ESTIMATE) algorithm (a method provided by Yoshihara et al.) [11]. Then we screened out 17 differentially expressed genes (DEGs) associated with immune score and stromal score. Based on these DEGs, 3 small molecule drugs were obtained, which showed strong potential for ccRCC treatment. Finally, transforming growth factor beta induced (TGFBI) was screened out by using four kinds of survival analyses and two independent datasets, which were significantly associated with prognosis of patients with ccRCC. 9 different kinds analyses were further performed to explore the potential value of TGFBI in various aspects.

In conclusion, our finding indicated that TGFBI had great effects for assessing prognosis of patients with ccRCC, which might be a novel prognostic biomarker and target for immunotherapy. Moreover, three molecule drugs were screened out, which might be novel choice for clinicians for ccRCC treatment.

## RESULTS

### Immune scores and stromal scores were correlated with clinical features of patients with ccRCC

Among all the 530 ccRCC downloaded from TCGA database, 35.1% (n = 186) samples were female, 64.9% (n = 344) samples were male. As for the neoplasm histologic grade, patients with Gx, G1, G2, G3, G4 grade accounted for 0.9% (n = 5), 2.7% (n = 14), 43.1% (n = 227), 39.1% (n = 206), 14.2% (n = 75), respectively. Tumors on the left accounted for 47.1% (n = 249) and the right side accounted for 52.9% (n = 280). Pathologic stage included 265 (50.0%) patients of stage I, 57 (10.8%) of stage II, 123 (23.2%) patients of stage III, and 83 (15.7%) patients of stage IV. When talking about person neoplasm cancer status, patients with tumor free accounted for 68.9% (n = 354) of the total number, and 31.1% (n = 160) cases were with tumor. After calculating immune score and stromal score of each ccRCC from TCGA-KIRC, immune scores ranged from -1109.36 to 2920.81 meanwhile stromal scores ranged from -1530.11 to 1955.39 as the result suggested (Supplementary Table 1).

**Figure 1:**
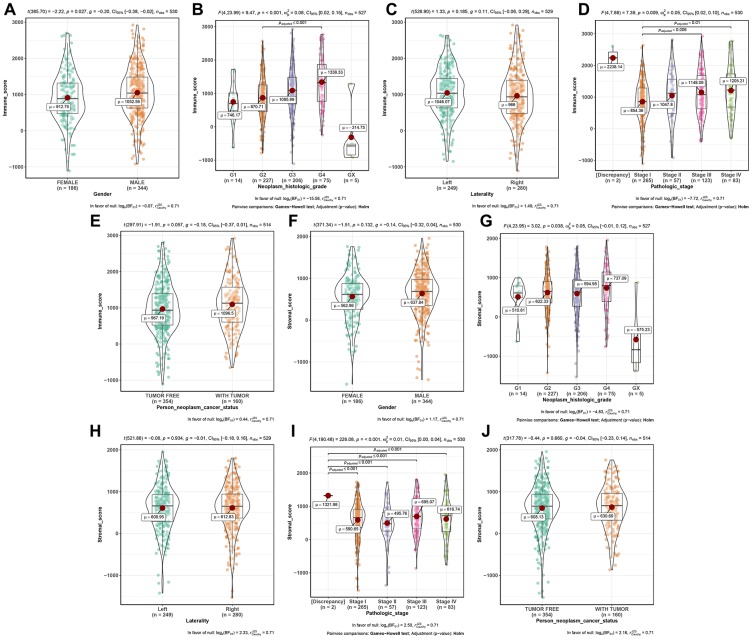
Distribution of immune scores of gender (**A**), neoplasm histologic grade (**B**), laterality (**C**), pathologic stage (**D**), and person neoplasm cancer status (**E**). Distribution of stromal scores of gender (**F**), neoplasm histologic grade (**G**), laterality (**H**), pathologic stage (**I**), and person neoplasm cancer status (**J**).

Further analysis demonstrated that immune score was associated with gender (*t* = -2.220, *P* = 0.027, Figure 1A), neoplasm histologic grade (*F* = 9.470, *P* < 0.001, Figure 1B), pathologic stage (*F* = 7.390, *P* = 0.009, Figure 1C), significantly. Moreover, stromal score was significantly related to neoplasm histologic grade (*F* = 3.020, *P* = 0.038, Figure 1G), and pathologic stage (*F* = 226.080, *P* < 0.001, Figure 1I).

### Immune scores and stromal scores were correlated with OS

**Figure 2:**
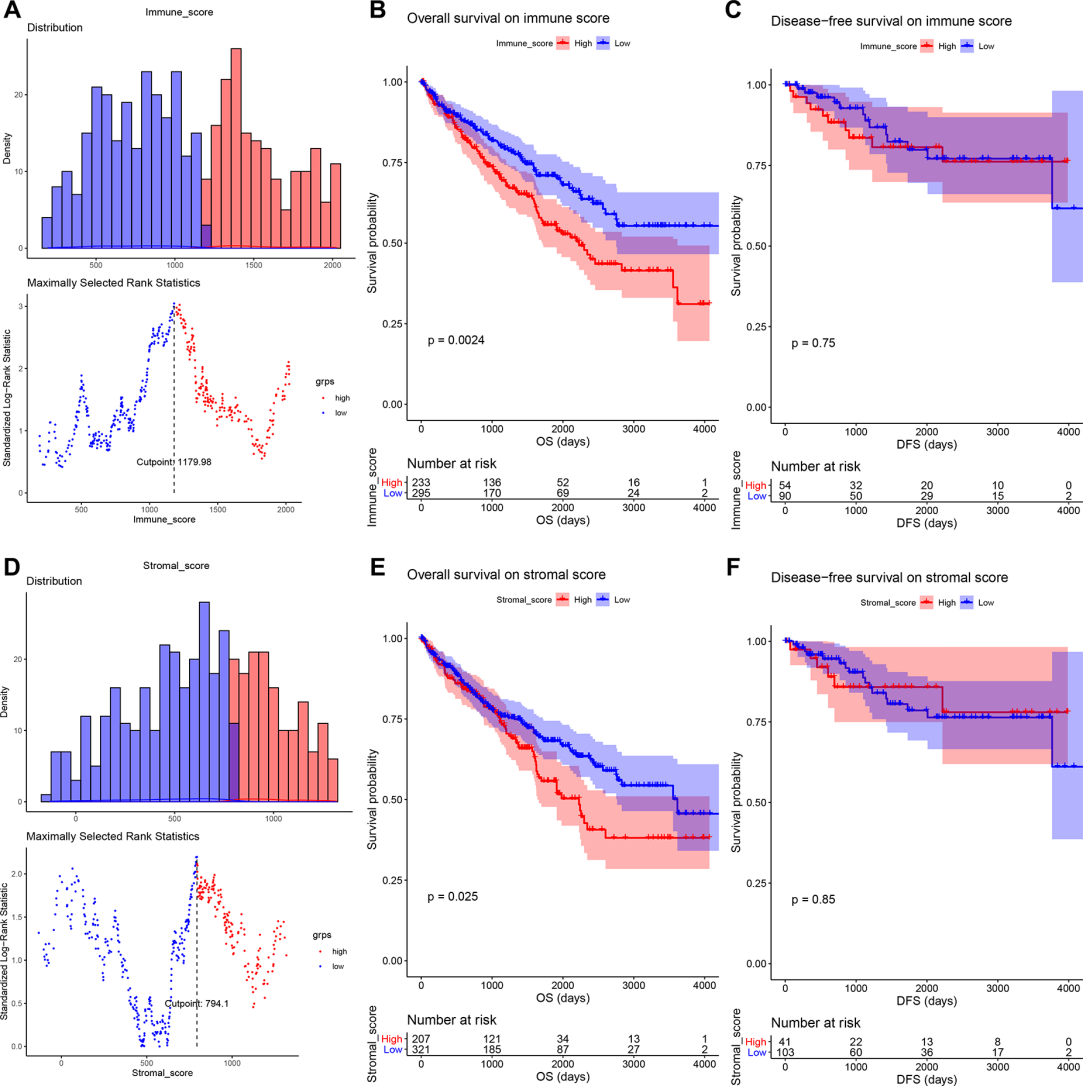
(**A**) An illustration of optimal cutoff identification for immune score. Survival analysis of the association between immune score and overall survival (**B**), disease-free survival (**C**) time in ccRCC. (**D**) An illustration of optimal cutoff identification for stromal score. Survival analysis of the association between stromal score and overall survival (**E**), disease-free survival (**F**) time in ccRCC.

To explore the relationship between immune score (or stromal score) and survival, we performed survival analysis in this part. As Figure 2A showed, 1179.98 was set as the optimal cutoff for grouping. ccRCC patients were divided into high immune score group (n = 234) and low immune score group (n = 296). The result suggested that ccRCC patients with high immune score had worse OS compared with these with low immune score (*P* = 0.0024, Figure 2B). Meanwhile, an illustration of optimal cutoff identification for stromal score is shown in Figure 2D (stromal score cutoff = 794.10). ccRCC patients were divided into two groups (high stromal score group: n = 208; low stromal group: n = 322). Similarly, low stromal score of cases was associated with better OS (*P* = 0.0250, Figure 2C). We also explored the correlation between scores and DFS, unfortunately, there was no significant relationship between them, as Figure 2C, 2F suggested.

### 17 DEG associated with immune score and stromal score screening

**Figure 3:**
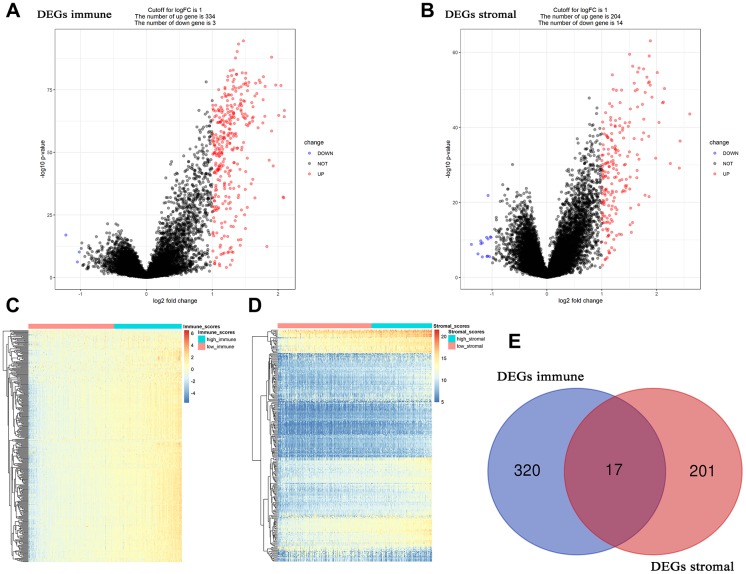
**Differentially expressed genes (DEGs) analysis in ccRCC.** (**A**) Volcano plot visualizing the immune-related DEGs. (**B**) Volcano plot visualizing the stromal-related DEGs. (**C**) Heatmap of immune scores of high score vs low score (*P* < 0.05, fold change > 1). (**D**) Heatmap of stromal scores of high score vs low score (*P* < 0.05, fold change > 1). (**E**) Identification of common DEGs between immune-related DEGs and stromal-related DEGs.

Based on “limma” in R software, 337 DEGs associated with immune score were screened out, including 334 up-regulated DEGs and 3 down-regulated DEGs (Figure 3A, 3C). Adjust *P* value and log2FC of each immune-related DEG were showed in Supplementary Table 2, in detail. Furthermore, 218 DEGs (204 up-regulated and 14 down-regulated) related to stromal score were picked out, accurately. Supplementary Table 3 showed the detailed information of each stromal-related DEG. Finally, 17 DEGs overlapped in immune-related DEGs and stromal-related DEGs were identified for further analysis (Figure 3E).

### Function and pathway enrichment analysis

**Figure 4:**
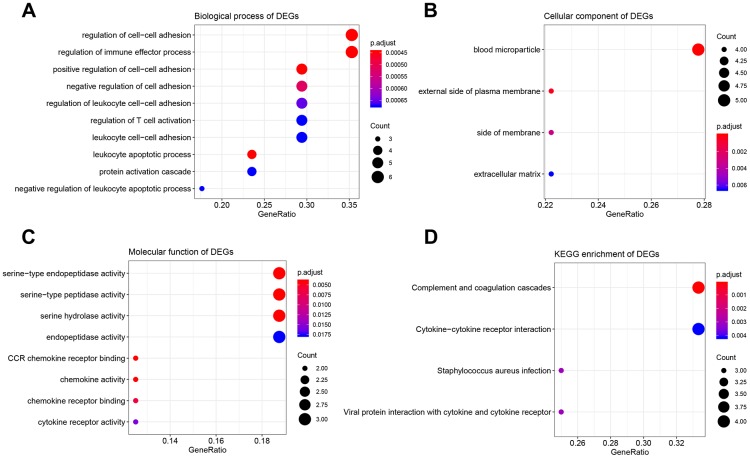
**Bioinformatics analysis of 17 DEGs associated with immune score and stromal score.** (**A**) Biological process of DEGs. (**B**) Cellular component of DEGs. (**C**) molecular function of DEGs. (**D**) KEGG enrichment of DEGs.

The results of GO analysis demonstrated that 17 DEGs were significantly enriched in 114 BPs (Supplementary Table 4), 4 CCs (Figure 4B), and 8 MFs (Figure 4C). The top 10 enriched BPs were regulation of cell-cell adhesion, regulation of immune effector process, leukocyte apoptotic process, positive regulation of cell-cell adhesion, negative regulation of cell adhesion, regulation of leukocyte cell-cell adhesion, regulation of T cell activation, leukocyte cell-cell adhesion, negative regulation of leukocyte apoptotic process, and protein activation cascade (Figure 4A). Moreover, DEGs were significantly correlated with four KEGG pathways including complement and coagulation cascades, cytokine-cytokine receptor interaction, staphylococcus aureus infection, and viral protein interaction with cytokine and cytokine receptor suggested by Figure 4D.

### 3 small molecule drugs might be novel choices for ccRCC treatment

The highly associated molecule drugs were identified by using CMap. Totally 6 molecule drugs were screened out (Table 1). Among them, three small molecule drugs including vincamine (mean = -0.493, n = 6, *P* < 0.001), clenbuterol (mean = 0.556, n = 5, *P* = 0.004), betazole (mean = 0.422, n = 5, *P* = 0.039) showed strong potential to treat ccRCC.

**Table 1:** Results of CMap analysis based on DEGs in ccRCC.

**cmap name**	**mean**	**n**	**enrichment**	**p**	**specificity**	**% non-null**
thioridazine	0.285	20	0.346	0.01256	0.6301	50
novobiocin	0.244	9	0.461	0.02866	0.0385	55
vincamine	-0.493	6	-0.742	0.00064	0.0059	66
myosmine	-0.256	6	-0.585	0.01788	0.0553	50
clenbuterol	0.556	5	0.724	0.00372	0	80
betazole	0.422	5	0.583	0.03915	0.0237	60

### Identification of 6 candidate hub genes

**Figure 5:**
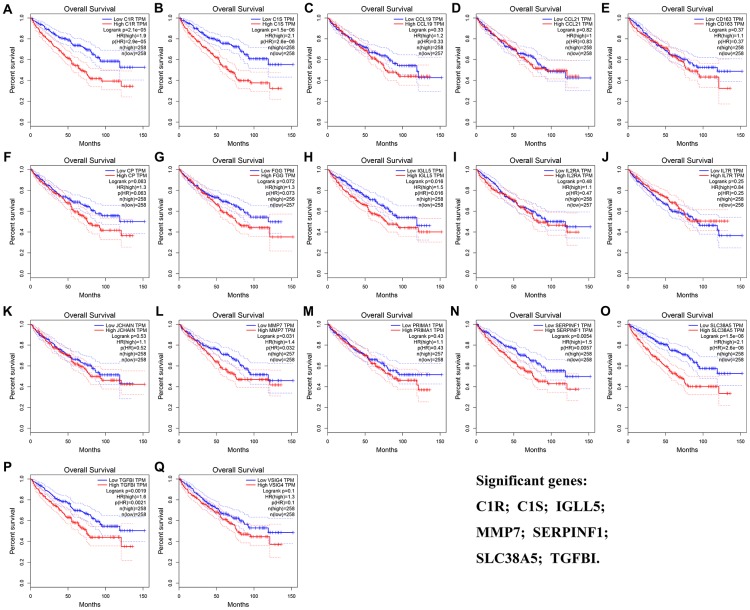
**Overall survival analyses on DEGs based on the TCGA-KIRC data.** (**A**) C1R. (**B**) C1S. (**C**) CCL19. (**D**) CCL21. (**E**) CD163. (**F**) CP. (**G**) FGG. (**H**) IGLL5. (**I**) IL2RA. (**J**) IL7R. (**K**) JCHAIN. (**L**) MMP7. (**M**) PRIMA1. (**N**) SERPINF1. (**O**) SLC38A5. (**P**) TGFBI. (Q) VSIG4.

**Figure 6:**
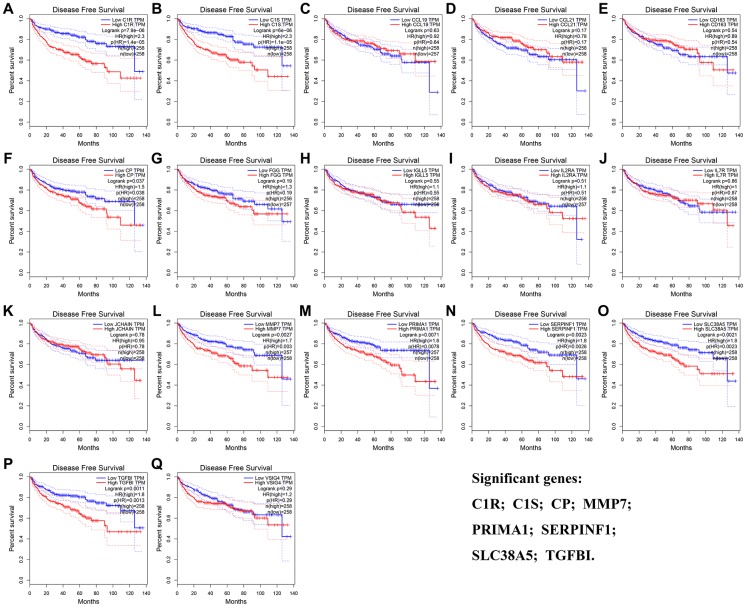
**Disease-free survival analyses on DEGs based on the TCGA-KIRC data.** (**A**) C1R. (**B**) C1S. (**C**) CCL19. (**D**) CCL21. (**E**) CD163. (**F**) CP. (**G**) FGG. (**H**) IGLL5. (**I**) IL2RA. (**J**) IL7R. (**K**) JCHAIN. (**L**) MMP7. (**M**) PRIMA1. (**N**) SERPINF1. (**O**) SLC38A5. (**P**) TGFBI. (**Q**) VSIG4.

The result of OS analysis demonstrated that 7 DEG including C1R (HR = 1.900, *P* = 2.1E-05, Figure 5A), C1S (HR = 2.100, *P* = 1.5E-06, Figure 5B), IGLL5 (HR = 1.500, *P* = 0.016, Figure 5H), MMP7 (HR = 1.400, *P* = 0.031, Figure 5L), SERPINF1 (HR = 1.500, *P* = 0.006, Figure 5N), SLC38A5 (HR = 2.100, *P* = 1.5E-06, Figure 5O), and TGFBI (HR = 1.600, *P* = 0.002, Figure 5P) were associated with OS of patients with ccRCC. In addition, high expressions of C1R (HR = 2.300, *P* = 7.9E-06, Figure 6A), C1S (HR = 2.300, *P* = 6E-06, Figure 6B), CP (HR = 1.500, *P* = 0.037, Figure 6F), MMP7 (HR = 1.700, *P* = 0.003, Figure 6L), PRIMA1 (HR = 1.600, *P* = 0.007, Figure 6M), SERPINF1 (HR = 1.800, *P* = 0.002, Figure 6N), SLC38A5 (HR = 1.800, *P* = 0.002, Figure 6O), and TGFBI (HR = 1.800, *P* = 0.001, Figure 6P) were significantly correlated with DFS of ccRCC patients. Finally, 6 genes including C1R (complement C1r), C1S (complement C1s), MMP7 (matrix metallopeptidase 7), SERPINF1 (serpin family F member 1), SLC38A5 (solute carrier family 38 member 5), and TGFBI (transforming growth factor beta induced) were selected as candidate hub genes for subsequent analysis.

### TGFBI was identified as hub gene

**Figure 7:**
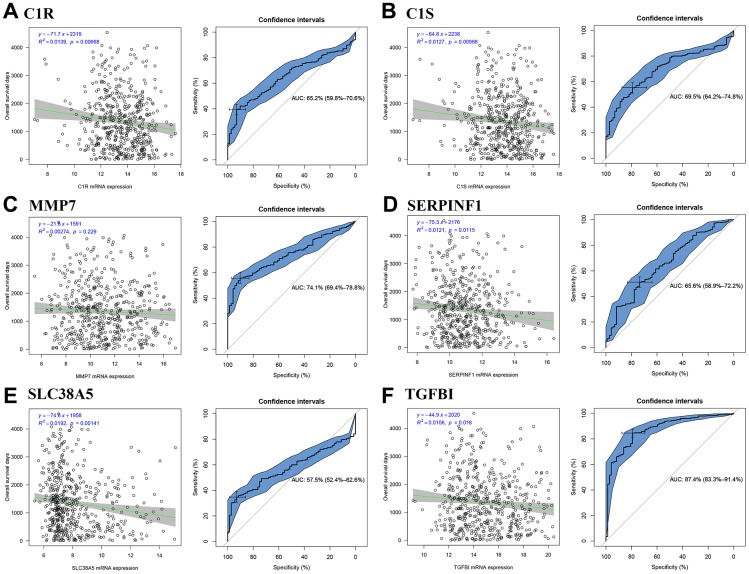
**Identification of hub genes.** Correlation between candidate hub genes and ccRCC patient overall survival days; ROC curve for candidate hub genes ((**A**) C1R; (**B**) C1S; (**C**) MMP7; (**D**) SERPINF1; (**E**) SLC38A5; (**F**) TGFBI).

We firstly calculated the R2 to evaluate the relationship between candidate hub genes and overall survival (OS) days. The result demonstrated that all the six genes (including C1R (R^2^ = 0.014, *P* = 0.007, Figure 7A), C1S (R^2^ = 0.013, *P* = 0.010, Figure 7B), SERPINF1 (R^2^ = 0.012, *P* = 0.012, Figure 7D), SLC38A5 (R^2^ = 0.019, *P* = 0.001, Figure 7E), and TGFBI (R^2^ = 0.011, *P* = 0.018, Figure 7F)) except MMP7 (R^2^ = 0.003, *P* = 0.229, Figure 7C) were negatively associated with OS days. Then we calculated AUC for each candidate hub genes. Only TGFBI reached the standard of AUC ≥ 0.80 (AUC = 0.874, Figure 7F), which was regarded as hub gene in this study.

### Patient living days gradually decreased with an increasing TGFBI expression

**Figure 8:**
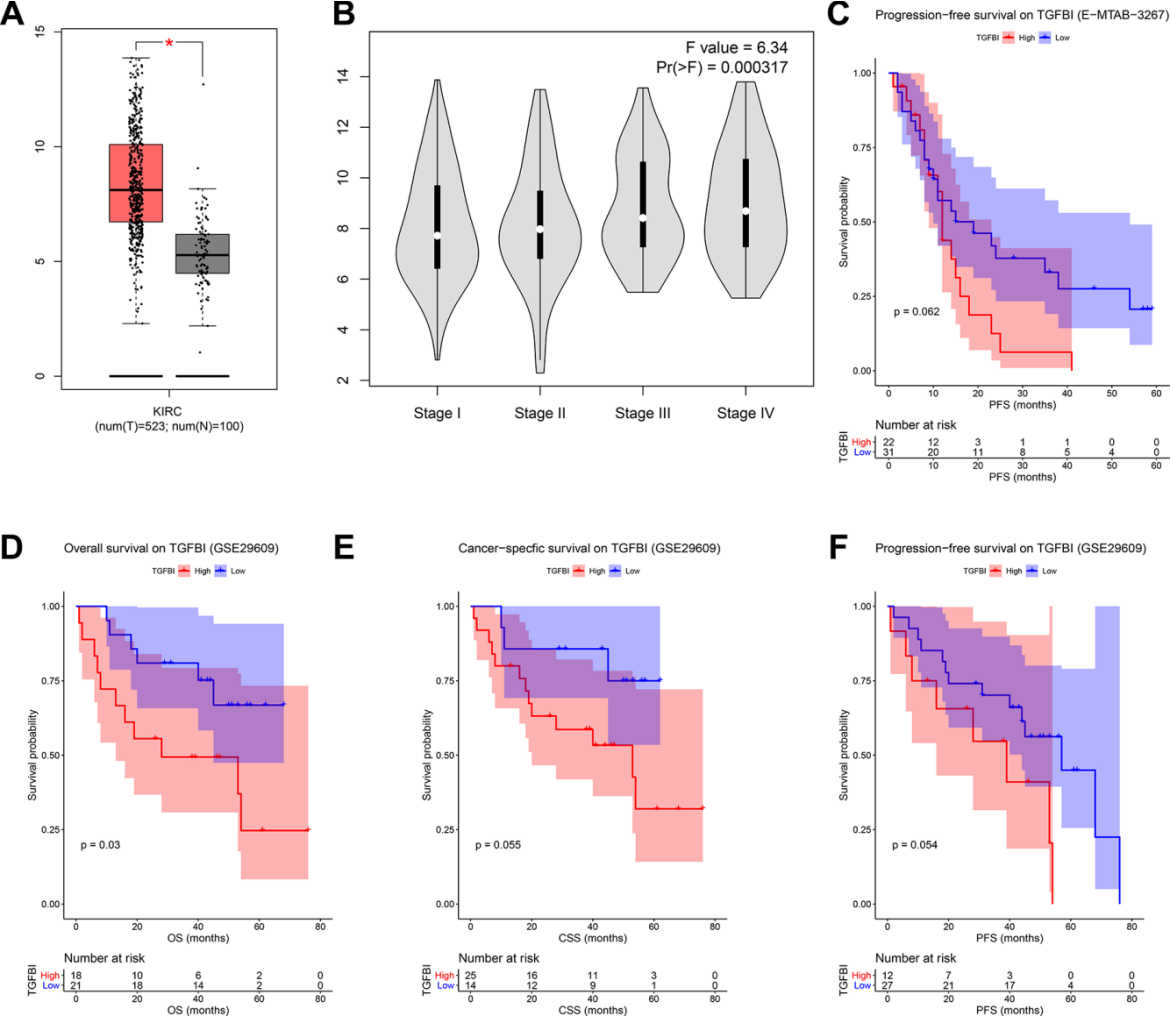
(**A**) Expression comparison of hub gene in ccRCCs and normal tissues by GEPIA. (**B**) Stage plot of TGFBI across different pathological stages in the TCGA-KIRC data. (**C**) Survival analysis of the association between TGFBI expression and progression free survival time in ccRCC (E-MTAB-3267). Survival analysis of the association between TGFBI expression and overall survival (**D**), cancer specific survival (**E**), progression free survival (**F**) time in ccRCC (GSE29609).

We firstly compared the mRNA expression levels of TGFBI between tumors and normal tissues, the result suggested that expressions of TGFBI in tumors were significantly higher than these in normal tissues (Figure 8A). Moreover, Expression of TGFBI (*F* = 6.34, *P* = 3.17E-04, Figure 8B) was significantly associated with tumor stage. High expression of TGFBI always related to higher tumor stage, as Figure 8B showed.

To validate the prognostic value of TGFBI, we performed three different survival analyses (OS, CSS, PFS) for all the candidate hub genes based on GSE29609. As shown in Figure 8D, high expressions of TGFBI (*P* = 0.030) were associated with worse OS. Meanwhile, there was a trend that patients with high expression of TGFBI had worse CSS (*P* = 0.066, Figure 8E) and PFS (*P* = 0.062 suggested by E-MTAB-3267 (Figure 8C), *P* = 0.054 suggested by GSE29609 (Figure 8F)). Summary above, we thought higher TGFBI expression was significantly associated with a shorter survival time, which showed great prognostic value in ccRCC.

### Hub gene validation

**Figure 9:**
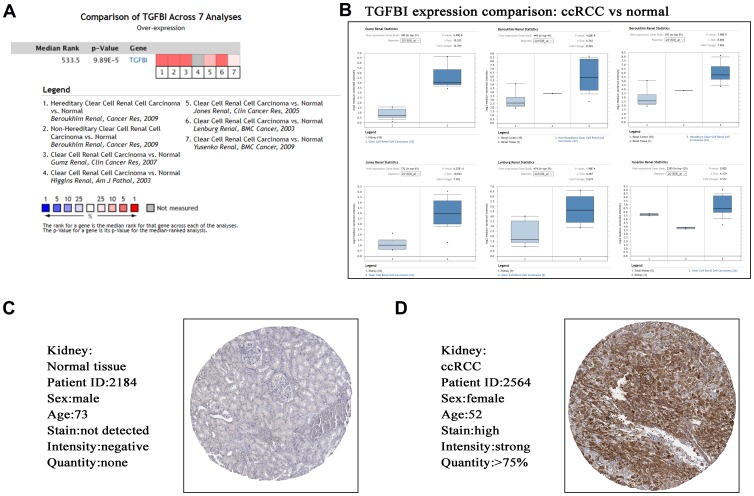
Validation of hub gene (TGFBI) in mRNA level (**A**, **B**) by Oncomine database and translational level (**C**, **D**) by The Human Protein Atlas database (IHC).

Based on ccRCC data from Oncomine database, TGFBI expression comparison between tumors and normal tissues across 7 analyses were identified. The result demonstrated that the mRNA expression of TGFBI (*P* = 9.89E-05, Figure 9A, 9B) was lower in normal tissues compared with ccRCCs. Furthermore, we explored the translational-level expression of hub genes. As Figure 9C, 9D showed, the translational-level expressions of TGFBI were higher in ccRCCs compared with normal tissues.

### mRNA expressions of TGFBI in cancer cell lines

**Figure 10:**
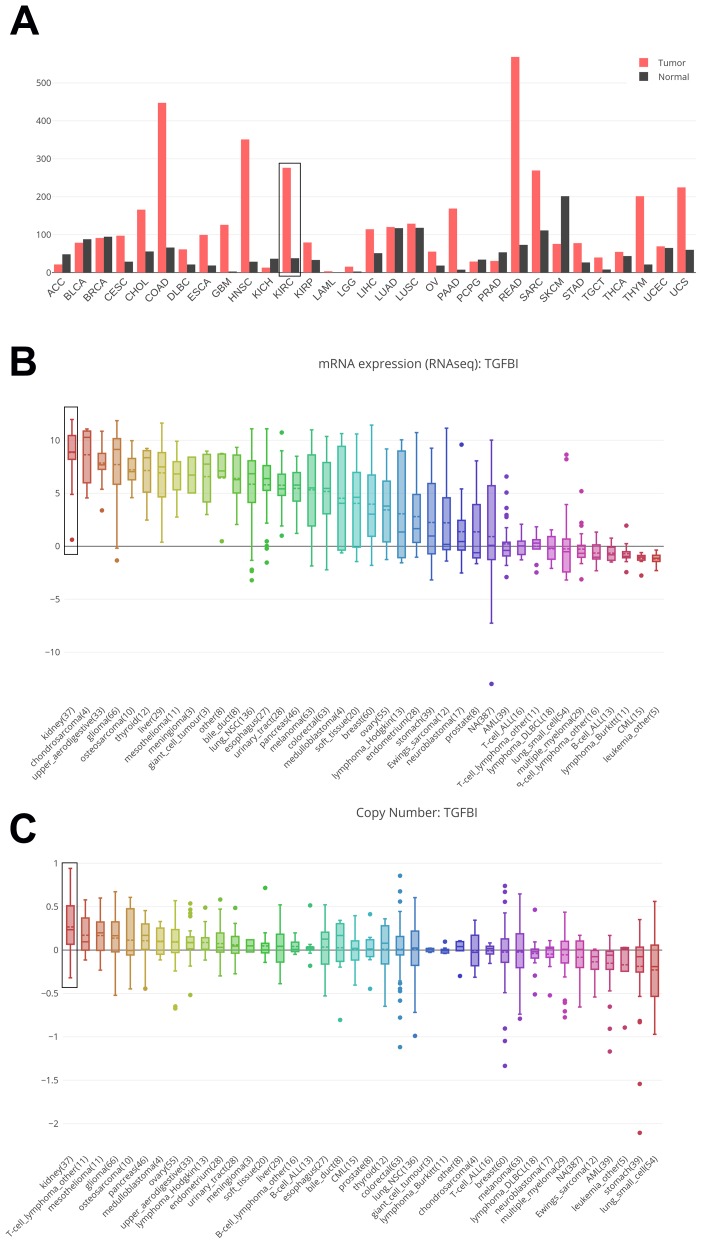
Comparison of TGFBI mRNA expression between tumors and normal tissues across all the types of cancers from TCGA data (**A**). Cancer Cell Line Encyclopedia analysis of TGFBI mRNA expression (**B**) and copy number variation level (**C**) in kidney (black boxes) and other cancer cell lines.

Based on GEPIA and CCLE, we explored hub genes expressions in all types of cancer cell lines. The expressions of TGFBI were different in these types of cancers. The expression of TGFBI was higher in GBM, HNSC, KIRC, KIRP, LGG, PAAD, SARC, STAD compared with corresponding normal tissues (Figure 10A). Moreover, we found that not only the mRNA expression (Figure 10B) but also the CNV level (Figure 10C) of TGFBI ranked as the first highest of all types of cancer cell lines. All the results made the hub genes reliable.

### Genetical alteration of TGFBI

**Figure 11:**
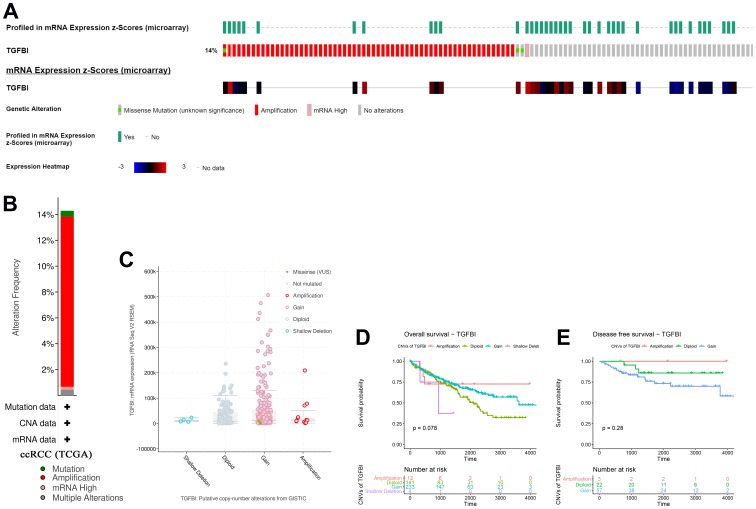
**A summary of mutations and CNVs of hub genes.** (**A**) Genetic alterations associated with hub genes and expression heatmap of hub genes based on the data from TCGA. (**B**) The total alteration frequency of TGFBI in TCGA-ACC is illustrated. Correlation between different CNV patterns and mRNA expression levels of TGFBI (**C**), respectively. (**D**, **E**) Survival analysis of ccRCC patients with CNVs of TGFBI based on TCGA-ccRCC data.

According to the result, TGFBI altered in 75 (14%) of 533 ccRCC patients (Figure 11A). And the main type was amplification (Figure 11B). Meanwhile, there was no differences of TGFBI expression between cases with CNVs and cases without CNVs (Figure 11C). When talking about the association between CNVs of TGFBI and survival of patients with ccRCC, there was a trend that amplification of TGFBI caused better OS (Figure 11D) and DFS (Figure 11E) of patients with ccRCC.

### Associations between TGFBI expression and clinical factors

Cases with complete clinicopathological data from TCGA database were included for this analysis. The result demonstrated that TGFBI expression was significantly associated with stromal score (*P* < 0.001), immune score (*P* < 0.001), gender (*P* < 0.001), neoplasm histologic grade (*P* < 0.001), pathologic stage (*P* = 0.011), and person neoplasm cancer status (*P* < 0.001) (Table 2).

**Table 2:** Associations between TGFBI expression and clinicopathological factors of patients with ccRCC (based on TCGA-KIRC).

**Characteristics**	**TGFBI expression**		**Chi-square/F**	**P**
	**Low**	**High**		
Stromal score				
Low	177	88	59.781	<0.001
High	88	177		
Immune score				
Low	166	99	33.879	<0.001
High	99	166		
Age				
≤65	164	169	0.202	0.653
>65	101	96		
Gender				
Male	148	196	19.085	<0.001
Female	117	69		
Laterality				
Left	122	127	0.227	0.634
Right	143	137		
NA		1		
Grade				
GX	4	1	24.392	<0.001
G1	10	4		
G2	125	102		
G3	103	119		
G4	20	55		
NA	3	0		
Stage				
Stage I	148	117	11.178	0.011
Stage II	32	25		
Stage III	53	70		
Stage IV	32	51		
Discrepancy				
Cancer status				
Tumor free	194	160	14.169	<0.001
With tumor	59	101		
NA	12	4		

### Prognostic value of TGFBI

**Figure 12:**
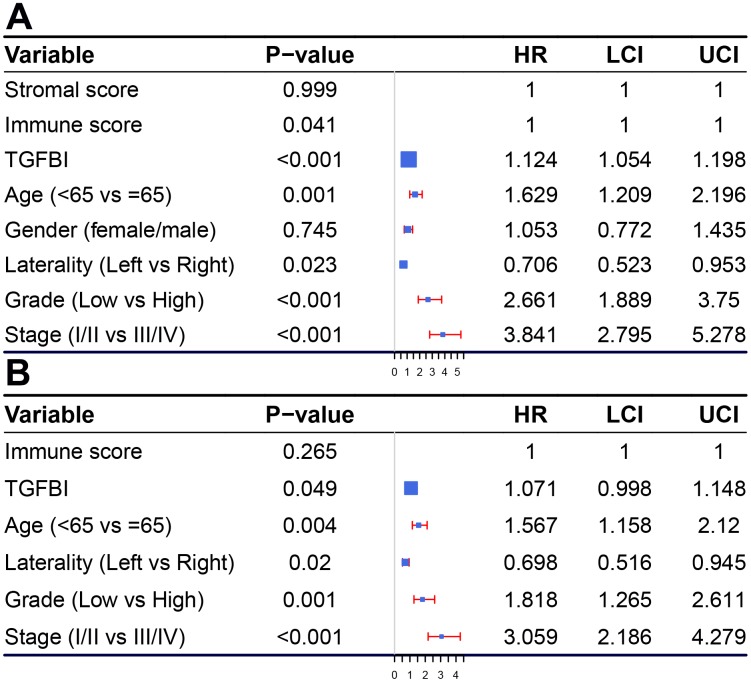
**Forest plot summary of analyses of OS.** Univariable (**A**) and multivariable analyses (**B**) of the stromal score, immune score, TGFBI, age, gender, grade, laterality, and tumor stage on all 530 patients.

According to the result (Figure 12A), immune score (Hazard Ratio = 1.000, 95%CI of ratio: 1.000-1.000, *P* = 0.041), TGFBI (Hazard Ratio = 1.124, 95%CI of ratio: 1.054-1.198, *P* < 0.001), age (Hazard Ratio = 1.629, 95%CI of ratio: 1.209-2.196, *P* = 0.001), laterality (Hazard Ratio = 0.706, 95%CI of ratio: 0.523-0.953, *P* = 0.023), neoplasm histologic grade (Hazard Ratio = 2.661, 95%CI of ratio: 1.889-3.750 *P* < 0.001), and pathologic stage (Hazard Ratio = 3.841, 95%CI of ratio: 2.795-5.278, *P* < 0.001) were influence features of OS as suggested by univariate Cox analysis. Even being adjusted by other features, TGFBI (Hazard Ratio = 1.071, 95%CI of ratio: 0.998-1.148, *P* = 0.049) was still relevant to OS among patients with ccRCC suggested by multivariate Cox analysis (Figure 12B).

### TGFBI expression was correlated with immune infiltration level in ccRCC

**Figure 13:**
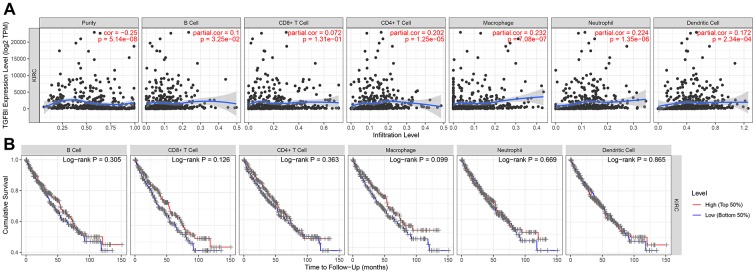
**Correlation of TGFBI expression with immune infiltration level in ACC.** (**A**) TGFBI expression was negatively relevant to tumor purity and positively related to macrophages. (**B**) Survival analyses across the six different tumor-infiltrating immune cells including B cells, CD8+ T cells, CD4+ T cells, macrophages, neutrophils, and dendritic cells.

Immune infiltration level played important role in survival in tumors. Therefore, we explored the relationship between hub genes and immune infiltration level. We found that TGFBI expression was negatively relevant to tumor purity (cor = -0.250, *P* = 5.14E-08) and positively related to macrophages (cor = 0.232, *P* = 7.08E-07), Figure 13A). Summary above we found TGFBI expression was significantly correlated with tumor purity in ccRCC, which suggested that TGFBI played an important role in immune infiltration in ccRCC.

### TGFBI might be potential DNA methylation biomarkers for ccRCC

**Figure 14:**
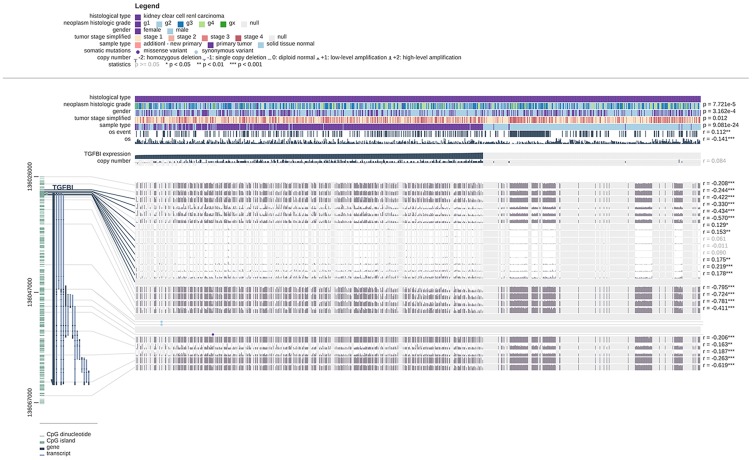
**Visualization of the TCGA data for TGFBI in ccRCC using MEXPRESS.** Samples were ordered by their expression value. This figure showed the correlation between hub gene expression and promoter methylation, with the Pearson correlation coefficients on the right (* p < 0.05, ** p < 0.01, *** p < 0.001).

We also explored the relationship between hub genes and methylation in this study by using MEXPRESS. The result demonstrated that the promoter region of TGFBI (Figure 14) showed higher methylation levels in ccRCC compared with normal tissues.

### Hub genes related KEGG signaling pathways

The result of GSEA (Table 3) suggested that TGFBI was significantly associated with two KEGG signaling pathways including cytokine cytokine receptor interaction (nominal *P* = 0.006, |ES| = 0.623, gene size = 257 and FDR = 14.763%), and chemokine signaling pathway (nominal *P* = 0.018, |ES| = 0.604, gene size = 185 and FDR = 14.794%).

**Table 3:** Genet set enrichment analysis (GSEA) of C1S and TGFBI.

**Genes**	**NAME**	**SIZE**	**ES**	**NES**	**NOM p-val**	**FDR**
TGFBI	KEGG_CYTOKINE_CYTOKINE_RECEPTOR_INTERACTION	257	-0.62328	-1.58451	0.005976	0.14763
	KEGG_CHEMOKINE_SIGNALING_PATHWAY	185	-0.60441	-1.63432	0.017613	0.147942

## DISCUSSION

As the most common subtype in kidney cancer, ccRCC was difficult to be cured. Patients with ccRCC always had poor prognosis [6]. Because of the lack of effective therapy target and molecule drugs for ccRCC treatment, the objective of this study was to screen out novel prognostic biomarkers and small molecule drugs in ccRCC.

Tumor microenvironment (TME) was the internal environment of tumor cells producing and survival, which described the non-cancerous cells present in the tumor [12]. These cells included but not limited to fibroblasts, immune cells and cells that comprise the blood vessels [13]. Some proteins (such as these produced by all of the cells present in the tumor which support the growth of the cancer cells) were also included in TME [14]. Among all the non-cancerous cells, immune cells and stromal cells were two major types, which had been proved to be correlated with prognosis of cancers [14]. Thus, in this study, we firstly used ESTIMATE algorithm for immune score and stromal score calculation. Then we divided cases from TCGA database into two groups (high-immune score group, and low-immune score group/high-stromal score group, and low-stromal score group). DEGs between the two groups were selected. 337 immune-related DEGs including 334 up-regulated and 3 down-regulated and 218 stromal-related DEGs including 204 up-regulated and 14 down-regulated were identified. 17 DEGs overlapped in the two kinds of DEGs were identified for further analysis. 6 genes including C1R (complement C1r), C1S (complement C1s), MMP7 (matrix metallopeptidase 7), SERPINF1 (serpin family F member 1), SLC38A5 (solute carrier family 38 member 5), and TGFBI (transforming growth factor beta induced) were picked out as candidate hub genes by performing OS and DFS analyses based on TCGA-KIRC data. Then we picked out one hub gene (TGFBI) by calculating the R^2^ and AUC for each candidate hub gene, which were validated in mRNA level and translational-level. The prognostic value of TGFBI was validated by applying OS, CSS, and PFS analyses based on GSE29609 and E-MTAB-3267. In addition, high expression of TGFBI was correlated to higher tumor stage. The result of TGFBI genetical alteration demonstrated that hub gene altered in 75 of 533 ccRCC patients, and the main type was amplification. Further analysis suggested that TGFBI expression was associated with gender, neoplasm histologic grade, and pathologic stage. Cox regression analysis also indicated that TGFBI was influence feature of OS of patients with ccRCC.

Considering about that immune infiltration level were significantly correlated with survival in tumors, we explored the relationship between hub gene expression level and immune infiltration level in ccRCC. The result demonstrated that TGFBI expression was correlated with tumor purity in ccRCC, which indicated that TGFBI played an important role in immune infiltration in ccRCC. We also explored the correlation between TGFBI and methylation around in the promoter region. The methylation levels of the promoter region of TGFBI were higher in ccRCC compared with normal tissues.

According to the function analyses, 17 DEGs were significantly enriched in 114 BPs, 4 CCs (blood microparticle, external side of plasma membrane, side of membrane, and extracellular matrix) and 8 MFs (main type: serine hydrolase activity). The top 10 BPs were regulation of cell-cell adhesion, regulation of immune effector process, leukocyte apoptotic process, positive regulation of cell-cell adhesion, negative regulation of cell adhesion, regulation of leukocyte cell-cell adhesion, regulation of T cell activation, leukocyte cell-cell adhesion, negative regulation of leukocyte apoptotic process, and protein activation cascade. Summary above we found that these DEGs showed strong correlation with immune response and tumor immune microenvironment.

To provide clinicians some novel choice for ccRCC treatment, CMap analysis was performed and the result indicated that 3 small molecule drugs including vincamine, clenbuterol, betazole showed strong potential for ccRCC treatment.

A literature review for hub genes was carried out in order to understand hub genes deeper and better. TGFBI encoded an RGD-containing protein that binds to type I, II and IV collagens, which played an important role in the regulation of a variety of BPs [15]. Ozawa et al. found that TGFBI expression in esophageal squamous cell carcinoma was associated with poor prognosis and hematogenous metastasis recurrence [16]. Chen et al. demonstrated that TGFBI was an important factor in epithelial-mesenchymal transformation (EMT) and malignant progression of prostate cancer [17]. Combining the literature review with this study, we believed that TGFBI played an important role in many events during immune response and tumor immune microenvironment.

However, this study also had certain limitations. Firstly, CD8+ cells played key role in tumor elimination. But in this study, the result (showed in Figure 13B, panel 2) demonstrated that CD8+ T cell infiltration had no or little significant effect on cumulative survival. Perhaps because of the unreasonable grouping and the TCGA data itself. Therefore, we will use novel dataset for this analysis in the short future. Secondly, CSS and PFS analyses of hub genes not showed significant results based on GSE29609 as we expected. Perhaps because of the few numbers of cases in GSE29609, which is related to too few data sets and the data itself. Thirdly, though we designed this research well and used strict thresholds for each part in this study, we did not conduct experiments to verify the results. Therefore, we will explore the related mechanisms of TGFBI in ccRCC through in vivo and in vitro experiments in further analysis.

## CONCLUSIONS

In summary, we identified 17 DEGs associated with immune score and stromal score by using TCGA-KIRC data. TGFBI was screened out as hub gene calculating the R2 and AUC for each candidate hub gene, which was validated in mRNA level and translational-level. The prognostic value of TGFBI was validated by applying OS, CSS, and PFS analyses based on GSE29609 and E-MTAB-3267. Further analysis indicated that TGFBI might be a novel prognostic biomarker and therapy target for immunotherapy. In addition, three molecule drugs including vincamine, clenbuterol, and betazole were identified, which showed strong potential to treat ccRCC.

## MATERIALS AND METHODS

### Data collection and data preprocessing

Microarray data of ccRCC (TCGA-KIRC data) was downloaded from The Cancer Genome Atlas (TCGA) database (https://genomecancer.ucsc.edu/). After excluding unqualified samples, totally 530 ccRCC samples from ccRCC patients were used in this study, which contained complete clinical information. Moreover, GSE29609 [18] performed on GPL1708 was retrieved from Gene Expression Omnibus (GEO) database (http://www.ncbi.nlm.nih.gov/geo/). This dataset including 39 ccRCCs with complete clinical information. Furthermore, E-MTAB-3267 including 53 ccRCCs with complete survival information was retrieved from ArrayExpress database (https://www.ebi.ac.uk/arrayexpress/). Both the two datasets were used for validating our findings.

**Figure 15:**
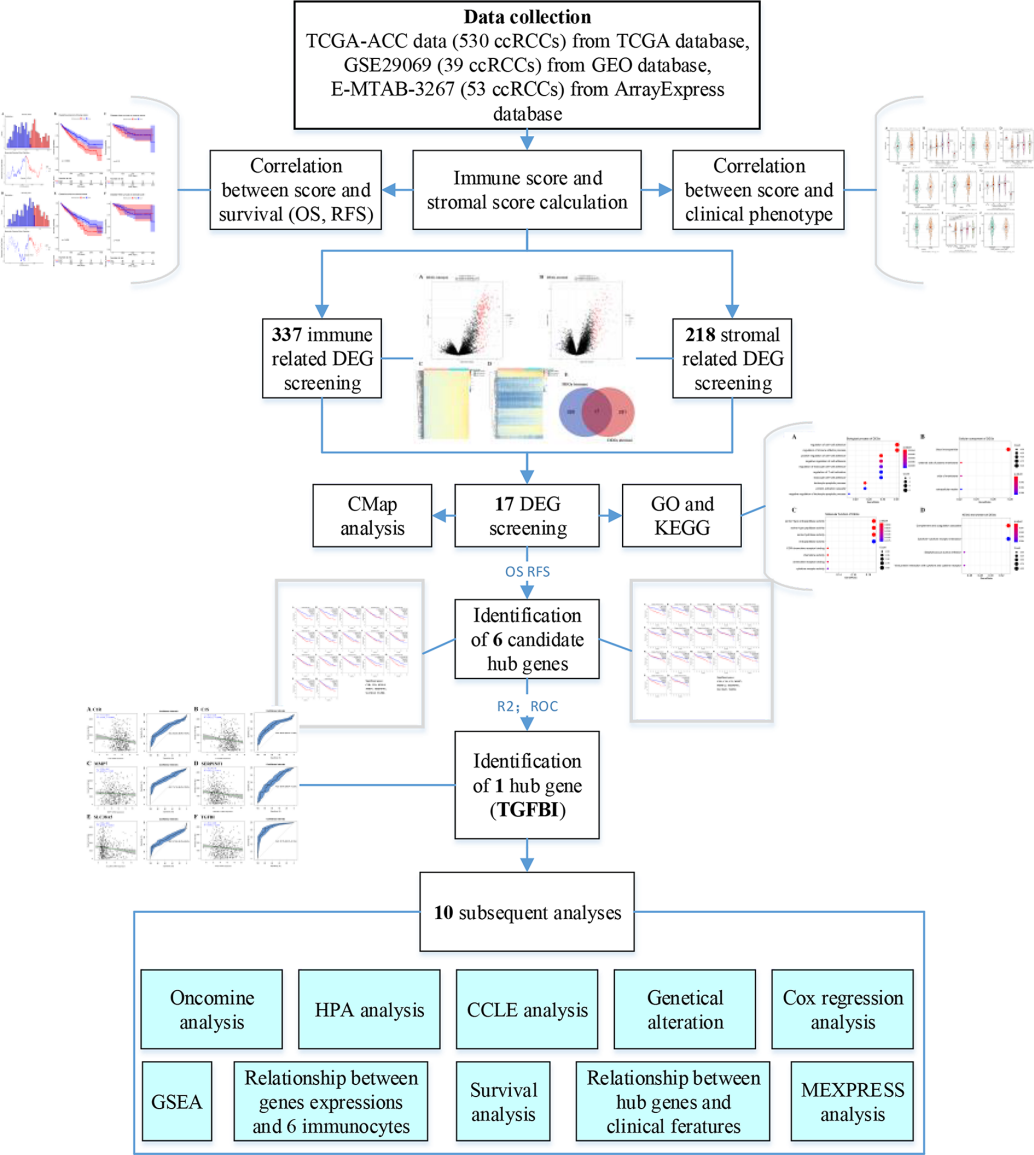
**Flow diagram of data preparation, processing, analysis, and validation in this study.**

Figure 15 showed the flow diagram of this study. For the TCGA-KIRC data displayed as counts number, normalized and log2 transformation were performed based on R package “DEseq.2” [19]. For GSE29069, the raw expression data was normalization and transformation by using R package “affy” [20]. For E-MTAB-3267, we directly downloaded the normalized expression matrix for ArrayExpress database.

### Immune score and stromal score calculation and their correlation with clinical phenotype

Based on TCGA-KIRC data, we firstly calculated immune score and stromal score of each sample by using ESTIMATE algorithm, which was applied by “estimate” [11] in R software. Then we compared the score difference between different gender (female vs male), neoplasm histologic grade (Gx, G1, G2, G3, G4), laterality (left vs right), pathologic stage (I, II, III, IV), and person neoplasm cancer status (tumor free vs with tumor), in order to explore the relationship between immune score or stromal score and important clinical phenotype. Moreover, survival analysis was performed to explore the correlation between immune score (or stromal score) and survival by using R package “survival” [21].

### Differentially expressed gene (DEG) screening

To pick out biomarkers associated with immune score and stromal score, we identified DEGs in this part. We firstly used R package “maxstat” for identifying the best score cutoff for grouping samples most significantly by applying a method called maximally selected rank statistics. To screen out DEGs related to immune score, we firstly divided 530 ccRCC samples into high-immune group and low-immune group based on the optimal immune score cutoff calculated by “maxstat”. Then “limma” [22] package in R was used for DEG screening. As for DEGs correlated to stromal score, we also divided 530 samples into two groups (high-stromal, and low-stromal) based on the optimal stromal score cutoff and screened out DEGs based on “limma” mentioned above. In this study, genes with Adjust *P* value < 0.05 and |log2 fold change (FC) | ≥ 1.0 were regarded as DEGs. The DEGs which exhibited the same expression trends in immune related DEGs and stromal related DEGs were picked out for further analysis.

### Function enrichment analysis

To explore potential functions of DEGs, Gene Ontology (GO) [23] enrichment analysis and Kyoto encyclopedia of Genes and Genomes (KEGG) [24] pathway analysis were performed through “clusterProfiler” [25] in R software. Gene sets were regarded as significantly enriched gene sets when P < 0.05.

### Candidate molecule drug identification

Connectivity map (CMap) [26] could help researchers to quickly identify molecule drugs with high correlation with diseases (https://portals.broadinstitute.org/cmap/). Thus, based on the DEGs we screened out before, we performed Connectivity map (CMap) analysis to explore molecule drugs (which had high correlation with ccRCC). In this study, drugs were thought to be highly correlated to ccRCC when number of instances (n) > 5 and *P* value < 0.05. Moreover, small molecule drugs with |mean| ≥ 0.40 were thought to have great potential for ccRCC treatment in this study.

### Candidate hub gene identification

In this study, we aimed to screen out some stromal-immune related prognostic biomarkers in ccRCC. Therefore, we performed two kinds of survival analyses (overall survival (OS) and disease-free survival (DFS)) for each DEG to screen out candidate hub genes based on Gene Expression Profiling Interactive Analysis (GEPIA) (http://gepia.cancer-pku.cn/) [27]. GEPIA collected 516 ccRCC samples with complete survival information, we divided 516 ccRCCs into high-expression group (n = 258) and low-expression group (n = 258) by evaluating the median value of each DEG. DEGs with *P* value < 0.05 in both the two survival analyses were thought as candidate hub genes.

### Hub gene identification

After screening out candidate hub genes, by using R package “basicTrendline” [28], we firstly calculated the R^2^ to see if candidate hub genes were associated with OS. *P* value < 0.05 was significant in this analysis. Furthermore, in order to evaluate the potential of distinguishing normal tissues and ccRCCs, we plotted receiver operating characteristic (ROC) curve and calculated AUC (area under curve) for each candidate hub gene (based on R package “pROC” [29]). In this study, we thought genes with *P* value < 0.05 in both the two analyses and AUC ≥ 0.80 were hub genes.

### Hub gene expressions comparison in tumors and normal tissues and in different pathologic stages

In this part, we firstly explore the expression levels of hub genes in tumors and normal tissues by using GEPIA. Unpaired t test was performed to measure the statistical significance. Based on TCGA-KIRC data with complete stage information, tumor stage (I, II, III and IV) boxplots were also performed. We used One-way Analysis of Variance (ANOVA) test to measure the statistical significance.

### Prognostic value of hub genes validation

Based on E-MTAB-3267 and GSE29609, three different survival analyses including OS, cancer specific survival (CSS), and progression free survival (PFS) were performed to validate the prognostic value of hub genes. *P* value < 0.05 was thought significantly.

### Oncomine analysis and translational-level expression validation

After screening out the hub genes, we assessed the mRNA expression levels of hub genes in ccRCC and normal tissue based on Oncomine database (https://www.oncomine.org/) [30]. Student t test was used to measure the statistical significance. Moreover, we validated the translation-level expression levels of hub genes by using The Human Protein Atlas database (https://www.proteinatlas.org/) [31].

### Cancer cell line encyclopedia (CCLE) analysis

To understand the hub genes better, we explored the mRNA expression levels and copy number variation (CNV) levels of hub genes in 40 types of tumors based on CCLE database (https://portals.broadinstitute.org/ccle/).

### Hub gene genetical alteration

To explore mutations and CNVs of hub genes, based on CBio Cancer Genomics Portal (http://www.cbioportal.org/) [32, 33], we first explored the genetic alterations of hub genes. Combined with relative mRNA expression levels of hub genes, the correlation between CNVs and mRNA expression levels of hub genes was also explored. Furthermore, we identified the relationship between CNVs and survival (OS, and DFS) of ccRCC patients by performing survival analysis.

### Exploring relationship between hub genes and clinical features of patients with ccRCC

Based on TCGA-KIRC data, we divided 530 ccRCCs into high-expression group (n = 265) and low-expression group (n = 265) by evaluating the median value of each hub gene expression. Then χ2 test or ANOVA was performed to analyze the correlations between hub gene expressions and clinical features (age, gender, laterality, neoplasm histologic grade, pathologic stage, and person neoplasm cancer status).

### Cox proportional hazards regression analysis

In this part, expression levels of hub genes and some important features (age, gender, laterality, neoplasm histologic grade, pathologic stage, and person neoplasm cancer status) were included for univariable Cox analysis of overall survival (OS) by using TCGA-KIRC data. Factors were included for multivariate Cox analysis when *P* value < 0.05. To do this, we could make sure if these hub genes were irrelevant to other clinical features for predicting OS of ccRCC.

### Exploring correlation between hub genes and immune microenvironment

By using TIMER (https://cistrome.shinyapps.io/timer/) [34], we explored the correlation between hub genes expressions and the abundance of immune infiltrates. Six kinds of tumor-infiltrating immune cells (B cells, CD8+ T cells, CD4+ T cells, macrophages, neutrophils, and dendritic cells) were included for this analysis [34]. Hub genes were thought to be significant associated with infiltrating level of an immunocyte when |correlation coefficient (cor) | ≥ 0.2 and *P* value < 0.05. Survival analysis was also performed between high infiltrating levels and low infiltrating levels of immunocyte.

### Exploring relationship between hub gene expression and methylation around in the promoter region

Considering about that MEXPRESS [35, 36] was a webtool for DNA methylation, expression, and clinical data visualization (https://mexpress.be/), we used this web tool to explore correlation between hub gene expression and methylation level. We calculated the Pearson correlation for difference (between expression value and methylation level) evaluation. For clinical factors contained two levels, P value was calculated to measure the difference. Furthermore, false discovery rate was calculated to compare the difference for clinical parameters which contained more than two levels.

### Gene set enrichment analysis (GSEA)

To explore the potential functions of hub genes, GSEA [37] was conducted by using TCGA-KIRC data. 530 ccRCCs were firstly divided into two groups (high-expression, and low-expression) based on the median of hub genes expression levels. We set “c2.cp.kegg.v7.0.symbols.gmt” as the reference gene sets. KEGG signaling pathways with nominal *P* < 0.05, |ES| > 0.6, gene size ≥ 100 and FDR < 25% were considered to be significantly enriched in this study.

## Supplementary Material

Supplementary Table 1

Supplementary Table 2

Supplementary Table 3

Supplementary Table 4
